# Does the spillage of petroleum products in *Anopheles *breeding sites have an impact on the pyrethroid resistance?

**DOI:** 10.1186/1475-2875-6-159

**Published:** 2007-12-03

**Authors:** Rousseau F Djouaka, Adekunle A Bakare, Honore S Bankole, Julien MC Doannio, Ousmane N Coulibaly, Hortense Kossou, Manuele Tamo, Harcourt I Basene, OK Popoola, Martin C Akogbeto

**Affiliations:** 1 International Institute of Tropical Agriculture, Cotonou, 08BP0932, Bénin; 2 Department of Zoology, University of Ibadan, Nigeria; 3 Université d'Abomey-Calavi, Bénin; 4 National Institute of Public Health, BPV47 Abidjan, Ivory Coast; 5 Ministry of Health, 05BP2099 Cotonou, Bénin; 6 Centre de Recherche Entomologique de Cotonou, 06 BP 2604, Bénin

## Abstract

**Background:**

The emergence of *Anopheles *populations capable of withstanding lethal doses of insecticides has weakened the efficacy of most insecticide based strategies of vector control and, has highlighted the need for further studies on the mechanisms of insecticide resistance and the various factors selecting resistant populations of mosquitoes. This research targeted the analysis of breeding sites and the oviposition behaviour of susceptible and resistant populations of *Anopheles *in localities of spilled petroleum products. The aim was to establish the possible contribution of oil spillage in the selection of pyrethroid resistance in malaria vectors.

**Methods:**

*Anopheles *breeding sites were identified and the insecticide susceptibility of the *Anopheles gambiae *populations mapped in 15 localities of South Western Nigeria. The presence of oil particles as well as the turbidity, the dissolved oxygen and the pH of each identified breeding site was recorded. Data were cross-analysed to correlate the habitat types and the insecticide susceptibility status of emerging mosquitoes. The second phase of this study was basically a laboratory model to provide more information on the implication of the spillage of petroleum on the selection of pyrethroid resistance in *An. gambiae*.

**Results:**

Moderate levels of resistance following exposure to permethrin-impregnated papers were recorded with the majority of *An. gambiae *samples collected in the South Western Nigeria. Data from this study established a link between the constituency of the breeding sites and the resistance status of the emerging *Anopheles*.

**Conclusion:**

This study has revealed the segregational occupation of breeding habitats by pyrethroid resistant and susceptible strains of *An. gambiae *in south-western Nigeria. Compiled results from field and laboratory research point out clear relationships between oil spillage and pyrethroid resistance in malaria vectors. The identification of this factor of resistance could serve as strong information in the management of insecticide resistance in some West African settings.

## Background

Malaria morbidity and mortality in tropical Africa remains high compared to other malaria-endemic areas of the world [1]. This is partially due to three efficient vectors of the subgenus Cellia: *Anopheles gambiae, Anopheles arabiensis *and *Anopheles funestus*. These species co-exist geographically across sub-Saharan Africa and can inhabit the same villages, shelter in the same houses, and feed on the same individuals [2]. Vector control strategies aim at reducing insect vector densities, vector life-span or to limit contact with humans with the final aim of reducing the transmission of diseases and, therefore, the related morbidity and mortality. These strategies are based on various methods including: physical, biological or chemical (such as insecticides) methods.

In the last decade in many African countries, the emergence of populations of *Anopheles *resistant to insecticides used in public health has been recorded [3,4]. This resistance phenomenon affects mainly the major vectors of malaria, *An. gambiae s.l*. [5] and *An*. *funestus s.l*. [6]. The resistance of *An*. *gambiae *to pyrethroids is documented in several parts of Africa [4,6-9] and has prompted several research activities into the underlying mechanisms of resistance [10] and factors contributing to the emergence of resistance [11]. According to Akogbeto *et al *[11], many mosquito species, *An. gambiae *in particular, lay their eggs in breeding sites located around agricultural settings. These eggs undergo a selection pressure from agricultural pesticides, which leads to the emergence of resistant strains [11]. This evidence on the implication of agricultural breeding sites in the selection of resistance, coupled with recent studies conducted in the Republic of Benin on the identification of petroleum concentrations likely to select resistance in *Anopheles *species [12], has guided this study. It was hypothesized in this study that spillage of petroleum products in mosquito breeding sites is probably implicated in the emergence of insecticide resistance in Nigeria. This hypothesis prompted the design of this field and laboratory study which focused on mapping, describing and analysis of the physico-chemical properties of water points yielding resistant and susceptible populations of *An. gambiae *in south-western Nigeria. In the laboratory, the oviposition behaviours and the larval growth of resistant and susceptible populations of *An. gambiae *from the field were monitored in reconstituted oily and non oily breeding sites. The identification of this factor of resistance could serve as strong indicator to help in the management of insecticide resistance in some West African settings.

## Methods

### Breeding sites identification and mapping, collection of *Anopheles *larvae

**Figure 1:**
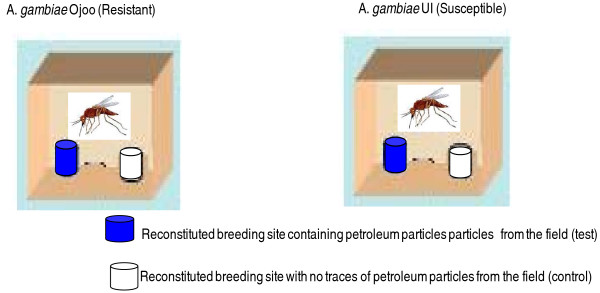
Oviposition preference of resistant and susceptible populations of *Anopheles *from the field.

**Figure 2:**
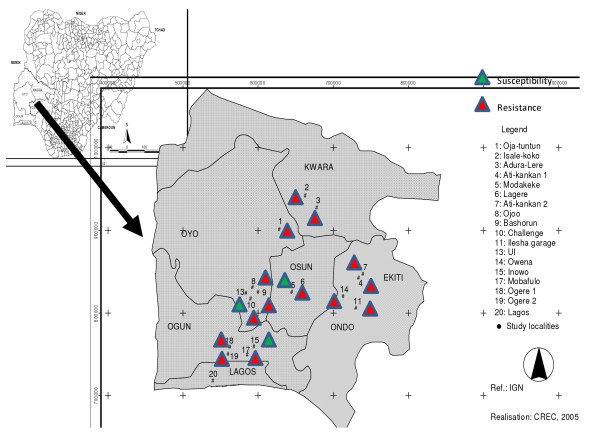
Mapping of *An. gambiae *susceptibility to pyrethroid (permethrin) in the South Western Nigeria.

This study was conducted in the south-western region of Nigeria. Samples were collected from five states in the region namely Ogun, Oyo, Osun, Ondo and Ekiti states (Figure 1). Standing water points found in each locality were systematically scrutinized for mosquito larvae. *Anopheles *larvae were recognized by their morphology and their horizontal position on water surface [13]. Larvae were sieved, concentrated in small buckets, washed with clean water and taken to the insectary of the Department of Zoology, University of Ibadan, Nigeria, where they were reared to adult stage for WHO bio-assays [14]. A Global Positioning System (GPS) device was used to record the latitude and longitude of each identified breeding site. These parameters were used for accurate positioning of surveyed sites on a map (Figure 2).

### Physico-chemical properties of breeding sites

Water samples collected from each identified breeding site were macroscopically described, and their pH and dissolved oxygen analyzed *in situ *using the multi-meter (Consort C535, Multi-variates). Macroscopical parameters considered in this study include the presence of petroleum particles in the water, the thickness of the water and the presence of oil layer at the surface of the breeding sites.

### WHO susceptibility tests on adult *Anopheles *from the field

Two to five-day old *Anopheles *emerging from the identified breeding sites, were bio-assayed using discriminating doses of insecticides. Bio-assays were carried out following WHO protocols [14]. Females of *An. gambiae *were exposed for one hour to papers impregnated with diagnostic dosages of permethrin (0.75%). For each test, five test tubes were used: one for the control and four for exposed mosquitoes. Control tubes contained filter papers impregnated with silicon oil (insecticide carrier), whereas treated papers were impregnated with diagnostic doses of insecticide plus carrier. An average of twenty mosquitoes was introduced in each tube. After one hour, mosquitoes were transferred to holding containers and provided with cotton pads with 10% sucrose solution. Mortalities were recorded at 24 hours and the susceptibility status of the population was graded according to the WHO protocol [14].

### Molecular characterization of anopheles populations from studied sites: PCR-species, PCR-forms and PCR-Kdr

In each locality, 20–30 females *Anopheles *from WHO Bio-assays were analysed at molecular level. PCR-species [15] was conducted in all *Anopheles *to identify the various members of *An. gambiae *complex encountered in each site. The next set of PCR focused on molecular forms [16] and involved only *An. gambiae sensu stricto (s.s.) *from the previous screening. The PCR forms sub-grouped the *An. gambiae s.s*. into two molecular forms: *An. gambiae s.s. (M) *and *An. gambiae s.s. (S)*. The last series of PCRs was to assess the presence of the *kdr *mutations in *An. gambiae ss*. populations [17]

### Laboratory monitoring of the oviposition preference and larval development in oily breeding sites

#### Characterization of *An. gambiae *Ojoo

*An. gambiae *Ojoo was selected in the locality of Ojoo in Ibadan, Oyo state. This strain is resistant to pyrethroids (Table 1) with no exhibition of the *kdr *mutation. The mechanism of resistance identified in this strain is a non-*kdr *mechanism of resistance. It is conceivable that a series of elevated enzymatic activities are responsible for resistance in this population [18]. The oviposition behaviour as well as the larval development of Ojoo strain on oily breeding sites were monitored and analysed in the laboratory.

**Table 1:** Resistance status of *An. gambiae s.l*. in some parts of South Western Nigeria

**States**	**Sampling sites main (city/town)**	**No. An. Tested**	**Mortality Rates**	**Localities Codes**	**Status**
Oyo	Ojoo (Ibadan)	80	80%	8	Resistance
	Bashorun (Ibadan)	75	70%	9	Resistance
	UI (Ibadan)	80	100%	13	Susceptibility
	Challenge (Ibadan)	80	81%	10	Resistance
	Oja-tuntun (Ogbomoso)	83	81%	1	Resistance
Osun	Lagere (Ife)	78	94%	6	Resistance
	Modakeke (Ife)	80	97%	5	Susceptibility.
Ondo	Ilesha garage (Akure)	77	89%	11	Resistance
	Owena (Owena)	78	75%	14	Resistance
Ekiti	Ati-kankan1 (Ado-ekiti)	83	85%	4	Resistance
	Ati-kankan2 (Ado-ekiti)	80	88%	7	Resistance
Ogun	Inowo (Ijebu-ode)	76	96%	15	Susceptibility.
	Mobalufon (Ijebu-ode)	75	80%	17	Resistance
	Ogere1(Lagos-Ibadan road)	4	75%	18	Resistance
	Ogere2 (Lagos-Ibadan road)	40	88%	19	Resistance
Lagos	Larvae died on the road	0	-	-	-

#### Characterization of *An. gambiae *UI

*An. gambiae *UI was selected on the campus of the University of Ibadan, Ibadan in Oyo state. This strain is susceptible to pyrethroids with mortality rates equal to 100%. (Table 1). In the field, *Anopheles *UI were found breeding in relatively clean water bodies with no visible particles of oil. The oviposition behaviour as well as the larval development of *Anopheles *UI was analysed in oily breeding sites reconstituted in the laboratory.

#### Simulation of oily breeding sites in the laboratory

Water samples with visible residues of petroleum products were collected in the field and more precisely in areas with spilled petroleum products (the locality of Ojoo). These samples (crude samples from the field) were used in the laboratory for the simulation of breeding sites. "un-sieved or crude or oily sites" indicated in this study was directly reconstituted by putting two litres of the water from the field into laboratory containers.

#### Oviposition preference of insecticide resistant and susceptible strains of *An. gambiae *from the field

50 well fed females of *An. gambiae *ready to lay eggs were introduced into a cage provided with 2 types of breeding sites for their oviposition: a breeding site with petroleum products from the field and a breeding site with no trace of petroleum products (control) (Figure 1). This experiment was carried out separately with *An. gambiae *Ojoo and *An. gambiae *UI. Cages were made in 4 replicates resulting in a total of 200 females of each strain monitored during this evaluation.

The number of eggs laid by each strain of *Anopheles *in the breeding site with petroleum products was counted and compared with that in the breeding site with no petroleum products. Statistical comparisons conducted on data generated during this experiment led to determination of the oviposition preference of each species and to comment on the level of selection pressure exercised by oily breeding sites during oviposition.

#### Impact of oily breeding sites on the larval development and the selection of resistant populations of *Anopheles*

Two hundred eggs of *An. gambiae *were introduced into breeding sites containing two liters of water collected in areas of spilled petroleum products in the south-western Nigeria. This experiment was replicated four times. The hatching rate and the number of larvae getting to pupae stage was determined for each strain of *Anopheles *(Ojoo and UI). Control breeding sites using clean water from the field with no trace of petroleum products were also seeded with eggs of the same species and monitored in tandem with test breeding sites. The number of hatched eggs and the number of larvae progressing to the pupae stage in each type of breeding sites and for each strain of *Anopheles *were determined. Statistical comparisons were used to determine the impact of oil spillage in the development of larvae of *Anopheles *Ojoo and UI and to identify the level of selection pressure exercised by oily breeding sites during larval development.

#### Data analysis

Analysis using computer software (Excel, SPSS and EpiInfo6) were performed on sets of data gathered from surveyed localities. Parameters for analysis include: the full description of the breeding sites of *Anopheles*, their physico-chemical properties, the resistance status of each tested population of *Anopheles*. The cross-analysis of these parameters led to identifying the impact of the quality of breeding sites on the emergence of resistant populations of *Anopheles*. Statistical tests of comparison were used to highlight the oviposition preference and the level of selection pressure exercised by oily breeding sites during larval development.

## Results

### The susceptibility pattern of *An. gambiae populations *from the South Western Nigeria to pyrethroids (permethrin)

Following the exposure of females *An. gambiae *to permethrin impregnated papers, a good spread of resistance was recorded in all the five states surveyed. Out of the 15 tested populations of *An. gambiae s.l*., 12 populations were clearly resistant to permethrin, whereas three were within the range of susceptibility as defined by WHO standards [14].

In Oyo state, out of the five localities studied (Ojoo, Basorun, UI, Challenge and Oja-tuntun), resistance was clearly recorded at Basorun (70% mortality), Ojoo (80% mortality), Challenge (81% mortality) and Oja-tuntun (81% mortality). No case of resistance was recorded among *Anopheles spp*. from UI (100% mortality). In Osun state, susceptibility was recorded at Modakeke (97% mortality) and resistance at Lagere (94% mortality) localities.

In Ondo state, the *Anopheles *populations from the two localities surveyed: Ilesha garage (Akure) and Owena (Owena) were all resistant to permethrin (89% and 75%, respectively).

In Ekiti state, resistance was recorded with *Anopheles *from the two localities sampled; 85% mortality rate was recorded at Ati-kankan1 (Ado-ekiti) and 88% at Ati-kankan2 (Ado-ekiti).

In Ogun state, resistance was recorded at Ogere1 (75% mortality) and Ogere2 (88% mortality), situated on the Lagos-Ibadan express way and at Mobalufon (80% mortality) in Ijebu-ode. A susceptibility pattern was noticed at Imowo (96% mortality), locality of Ijebu-ode.

In most screened breeding sites, cadavers of larvae were recorded: probably due to lethal activities of petroleum products. The summary of the results of bio-assays is shown in (Table 1).

### Mapping of *An. gambiae *susceptibility to pyrethroids in studied localities

Using geographical information recorded with the GPS, localities screened were projected on a map of Nigeria and areas of permethrin resistance demarcated. The map generated from this study showed a spread of resistance in most states of South Western Nigeria. The levels of resistance registered in the localities of Basorun, Owena, and Ogere were relatively consistent compare to other sites. On the other hand, no resistance was found in UI. A reduced susceptibility to permethrin was recorded in Modakeke and Imowo (Figure 2).

### Impact of the quality of the breeding site on the susceptibility status of emerging *Anopheles*

Cross-analyses were made with data on the quality of the breeding sites (physico-chemical parameters, presence of petroleum particles) and the pyrethroid susceptibility status of emerging adults of *An. gambiae*. Very slight differences were obtained between the pH of breeding sites producing resistant populations of *An. gambiae *and those producing susceptible populations. Contrary to data on the pH, breeding sites having petroleum particles and therefore low levels of oxygen seemed to produce resistant strains of mosquito. It is possible that the survival rate of eggs laid by susceptible mosquitoes in oily breeding sites is low. This inhibition of eggs or larvae development (P = 0.0015) could explain the low presence of susceptible populations of *Anopheles *in oily breeding sites. In the three breeding sites with little or no oil particles (breeding sites No.13, 5, 15), a consistent number of pyrethroid susceptible populations of *An. gambiae *was recorded (Table 2 and 3).

**Table 2:** Physico-chemical properties of breeding sites producing susceptible and resistant populations of *An. gambiae*

Locality and Code	Susceptibility to Permethrin	Breeding site type	Oxygen levels in breeding sites (mg/l)	pH Level	Mortality rates of adults to Permethrin (%)
13	Susceptible	no oil	52.5	7.8	100% (n = 80)
5	Susceptible	no oil	35	7.0	97% (n = 80)
15	Susceptible	no oil	19.5	8.0	96% (n = 76)
6	Resistance	Oily	20	7.7	94% (n = 78)
11	Resistance	Oily	16.5	7.6	89% (n = 77)
7	Resistance	Oily	15	7.7	88% (n = 80)
19	Resistance	Oily	13	7.6	88% (n = 40)
4	Resistance	Oily	15	7.6	85% (n = 83)
10	Resistance	Oily	12	7.5	81% (n = 80)
1	Resistance	Oily	13	7.8	81% (n = 83)
8	Resistance	Oily	12	7.9	80% (n = 80)
17	Resistance	Oily	13	7.8	80% (n = 75)
14	Resistance	Oily	10	8.0	75% (n = 78)
18	Resistance	Oily	10	7.8	75% (n = 04)
9	Resistance	Oily	12	8.0	70% (n = 75)

**Table 3:** Comparison of the mean mortality rates when *Anopheles *from oily and non oily sites are exposed to permethrin

	**Oily breeding site**	**Non oily breeding site**
Number of breeding sites surveyed during the study	12	3
Number of tested females of *An. gambiae *from identified breeding sites	833	236
Mortality rates (mean) recorded with *Anopheles *from each type of breeding sites following exposure to permethrin	82.%	97.6%
Variance (mortality rates in surveyed localities)	4.33	47.7
Pv. Comparing the mean mortalities (following exposure to permethrin) recorded with mosquito emerging from oily and non oily breeding sites	**Pv = 0.00151**	

### Breeding sites preference by pyrethroid susceptible and resistant strains of *Anopheles*

**Figure 3:**
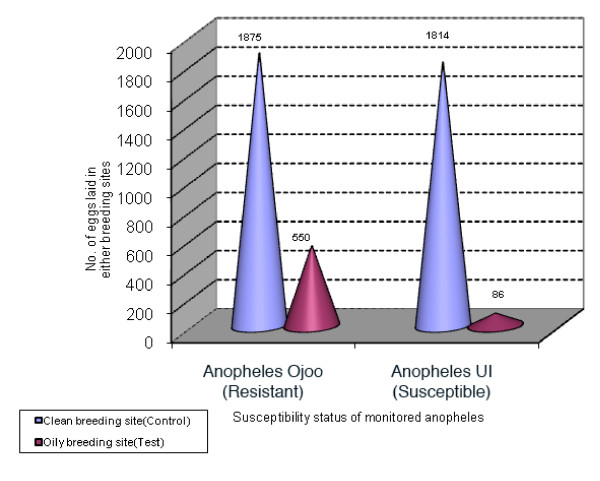
Number of eggs laid by susceptible and resistant strains of *Anopheles *in oily and non oily breeding sites.

In the laboratory, both pyrethroid susceptible and resistant strains of *Anopheles *exhibited a high reluctance for laying eggs in breeding sites containing petroleum products. Out of a mean number of 1,900 eggs laid in each cage by the susceptible strain *Anopheles *UI, 4.5% (86 eggs) of these eggs were deposited in oily breeding sites and 95.5% (1814 eggs) in the clean breeding site (control site). With the resistant strain *Anopheles *Ojoo, Out of a mean number of 2,425 eggs laid in cages, 77% (1875 eggs) of these eggs were deposited in breeding sites with clean water (control site) whereas 23% (550 eggs) of these eggs were laid in breeding site with petroleum debris (Figure 3). This partially reveals an acceptable level of tolerance of oily breeding sites by resistant females of *An. gambiae *compare to susceptible ones (UI/Ojoo, P = 0.00000). The very low tolerance to oily breeding sites by pyrethroid susceptible populations of *Anopheles *during oviposition translates the high selection pressure exercised on this mosquito species in localities where most breeding sites are covered with petroleum products.

### Hatching rate of eggs laid by pyrethroid susceptible and resistant strains in oily breeding sites

**Figure 4:**
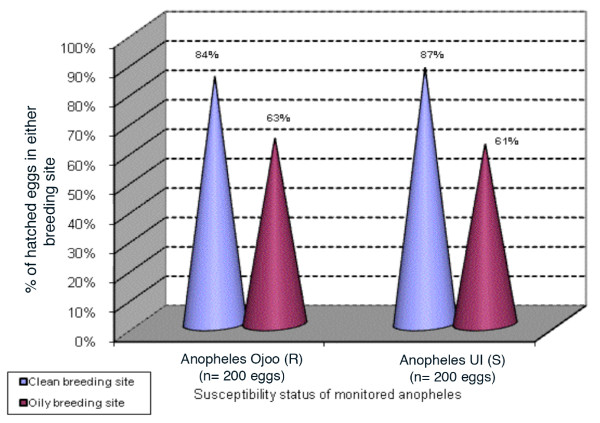
Hatching rate of eggs laid by resistant and susceptible strains of *Anopheles *in oily and non-oily breeding sites.

Of a total of 400 eggs of *Anopheles *UI monitored during this experiment, 200 eggs were introduced in breeding sites containing oil particles (test) and the remaining 200 in the breeding site with no oil (control). Results generated showed that 61% (122 eggs) of the 200 eggs hatched in oily breeding sites and 87% (174 eggs) of 200 eggs hatched in breeding site with no petroleum products (control). With the Ojoo strain, 84% (168 out of 200 eggs) hatching rate was recorded in the control breeding site whereas 63% (126 out of 200 eggs) hatching rate was found in the breeding site with oil products. These results point to a significant impact of oil products on the hatching of eggs of both tested strains but with a more pronounced hatching inhibition on the susceptible strain *Anopheles *UI (UI, P = 0.00000 and Ojoo, P = 0.0002) (Figure 4).

### Development of larvae of pyrethroid susceptible and resistant strains in oily breeding sites (rate of larvae getting to pupae stage)

**Figure 5:**
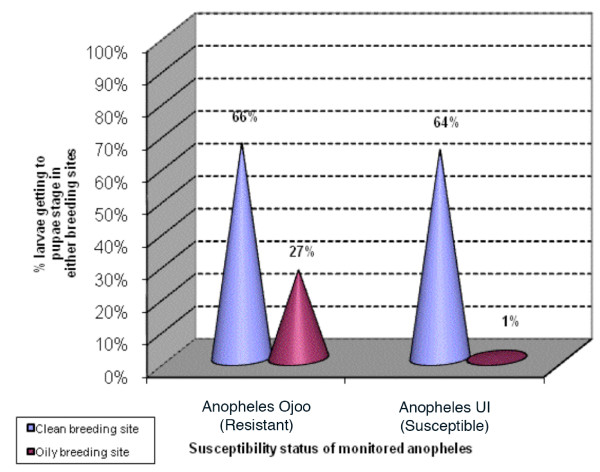
Rate of larvae (hatched lavae from eggs) getting to pupae stage during development in oily and non oily breeding sites.

A catastrophic effect of oil was recorded during the development of the susceptible larvae of *Anopheles *UI. Very few larvae of this strain (1%) could reach the pupae stage in breeding sites polluted with petroleum products (only 1 pupa from the 122 L1-larvae emerging from hatched eggs). With *Anopheles *Ojoo 27% (34 out of 126 hatched individuals) of larvae were able to reach the pupae stage when reared in water with oily debris. When the rearing was conducted in clean breeding sites (control breeding sites), we recorded a relatively high number of pupae from both strains (64% and 66% of L1-larvae emerging from hatched eggs got to the pupae stage in non oily breeding sites with the susceptible and resistant strain respectively) (Figure 5). Although a lethal effect of petroleum products was globally observed with both strains, the destructive impact of these products was more prominent on larvae of UI compare to those of Ojoo (UI/Ojoo, P = 0.00000). These results suggest a high selection pressure at larval stage of resistant populations of *Anopheles *by spilled petroleum products (Figure 5).

## Discussion

This study investigated the possible changes into *An. gambiae *populations when they are challenged with petroleum products. The main goal of this study was to identify the relationship between oil spillage and the emergence of pyrethroid resistance in malaria vectors in the South Western Nigeria. Results from insecticide bio-assays revealed a spread of pyrethroid resistance in the South Western Nigeria. The presence of *Anopheles *populations capable of withstanding diagnostic doses of permethrin was initially reported in this part of Nigeria [19,20].

**Figure 6:**
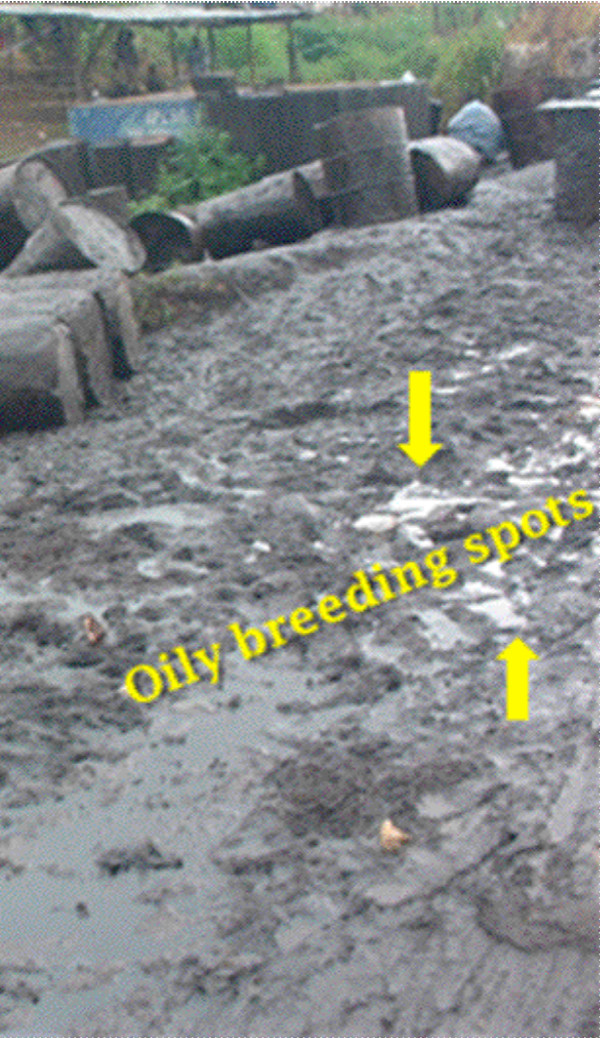
Spillage of petroleum products by oil retailers in the locality of Ojoo, Ibadan. Pointed in yellow are identified oily breeding sites yielding resistant populations of *An. gambiae*.

**Figure 7:**
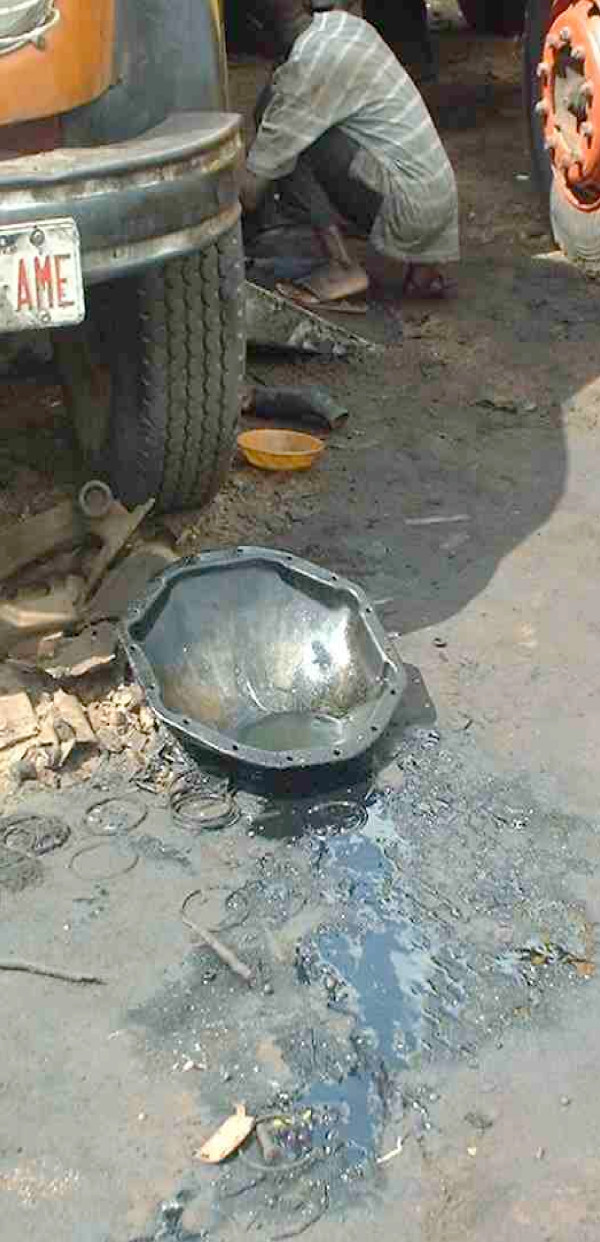
Spillage of petroleum products by mechanics.

The massive use of pesticides in agricultural settings has been well documented as a factor contributing to the emergence of resistance in *Anopheles *populations [11]. As reported by Akogbeto, some populations of *An. gambiae *lay their eggs in breeding sites containing insecticide residues. Larvae from these eggs are subjected to a selection pressure leading to the pyrethroid resistance [11]. It is possible that the presence of petroleum particles in breeding sites could have a similar activity in water bodies containing mosquito larvae. Ojoo and UI are close localities situated at less than 2 km from each other. At Ojoo, where most breeding sites are covered with petroleum, mosquitoes were mostly resistant, whereas at UI, very close sites to Ojoo, but with no oil spillage, most susceptible populations of mosquitoes collected here were found in the clean and non-oily sites. Petroleum products in *Anopheles *breeding sites probably exercise a severe selection pressure by killing most susceptible strains of *An. gambiae *and allowing the emergence of mainly resistant individuals as observed in the laboratory. Projected in the field, it is possible that this laboratory model could explain the absence of susceptible strains of *An. gambiae *in oily breeding sites and the predominance of resistant populations of *Anopheles *in areas where most breeding sites are covered by spilled petroleum products (Figure 6 and 7). This presumptive analysis could be corroborated with data from Ojoo and UI which are close localities (less than 2 km) but with different levels of susceptibility. These differences of susceptibility in both localities are probably justified by the nature of breeding sites. Also the molecular analysis of samples from UI and Ojoo did not show a significant variability in both populations (Table 4). The absence of the *kdr *mutations on samples analysed during this study suggests the selection of mechanisms different from *kdr *mutations by petroleum products. The use of petroleum products against malaria vectors has been reported in several communities in the Western part of Africa [12]. Similarly to other classical insecticides, it is also possible that petroleum products while killing a good number of mosquito larvae also allow the emergence of few ones which are cross resistant to pyrethroids.

**Table 4:** Molecular characterization of samples analysed during the study

**States**	**Sampling sites main (city/town)**	**Localities Codes**	**Status**	**PCR-Species (*n *= number of mosquito tested**	**PCR-form**	**PCR-kdr**
Oyo	Ojoo (Ibadan)	8	Resist	*An. gambiae ss (n = 30)*	M	No Kdr
	Bashorun (Ibadan)	9	Resist	*An. gambiae ss (n = 30)*	M	No Kdr
	UI (Ibadan)	13	Suscept	*An. gambiae ss (n = 30)*	M	No Kdr
	Challenge (Ibadan)	10	Resist	*An. gambiae ss (n = 30)*	M	No Kdr
	Oja-tuntun (Ogbomoso)	1	Resist	*Arabiensis (n = 30)*	-	
Osun	Lagere (Ife)	6	Resist	*An. gambiae ss (n = 30)*	M	No Kdr
	Modakeke (Ife)	5	Suscept	*An. gambiae ss (n = 30)*	M	No Kdr
Ondo	Ilesha garage (Akure)	11	Resist	*An. arabiensis (n = 30)*	-	
	Owena (Owena)	14	Resist	*An. arabiensis (n = 30)*	-	
Ekiti	Ati-kankan1 (Ado-ekiti)	4	Resist	*An. arabiensis (n = 30)*	-	
	Ati-kankan2 (Ado-ekiti)	7	Resist	*An. arabiensis s (n = 30)*	-	
Ogun	Inowo (Ijebu-ode)	15	Suscept	*An. arabiensis s (n = 30)*	-	
	Mobalufon (Ijebu-ode)	17	Resist	*An. arabiensis (n = 30)*	-	
	Ogere1	18	Resist	*An. arabiensis (n = 4)*	-	
	Ogere2	19	Resist	*An. arabiensis (20)*	-	
Lagos	Larvae died on the road	-	-	*Not tested*	-	

Nigeria is among the first oil producers in the world [21]. Oil dumping is an old and generalized practice in this country. The management of used petroleum products is a critical and very sensitive issue in this part of the world and leads to wide-spread environmental pollution and the development of various health hazards. Petroleum products are spilled on a wide-scale in the environment by mechanics, retailers, trailers and old cars. These products are washed by rains and accumulate in low-lying areas where, mixed with water, they constitute breeding spots for mosquitoes. *Anopheles *may lay their eggs in these oily breeding sites and selection pressures may be exercised during series of larval development cycles. A cross-resistance seems to exist between the capability to survive petroleum exposures and lethal doses of pyrethroids.

The molecular mechanism developed by populations of Anopheles against spilled petroleum products is unclear. Further investigations using advanced molecular techniques and technologies to investigate the molecular pathways involved in the emergence of pyrethroid resistance from series of exposures to petroleum products are needed. Microarray analyses of samples of *An. gambiae *from the South Western Nigeria using the "detox chip" are in progress at the Liverpool School of Tropical Medicine, UK. This may elucidate the gene expression profile of species of *Anopheles *selected with petroleum products and further contribute to highlighting the implication of petroleum products in the emergence of pyrethroid resistance in South Western Nigeria.

## Conclusion

This study analysed the potential impact of oily breeding sites in the development of both susceptible and resistant strains of *An. gambiae *and, has highlighted the potential contribution of spilled petroleum products in the development and spread of pyrethroid resistance in malaria vector in south-western Nigeria. Similar to the implication of agricultural pesticides in the emergence of pyrethroid resistance in mosquito populations, spilled petroleum products are washed by rains and accumulate in low-lying areas where, mixed with water, they constitute breeding spots for mosquitoes. *Anopheles *will lay their eggs in these oily breeding sites; both the eggs and the larvae emerging from hatched eggs will undergo series of selection pressure which ends with the emergence from these oily breeding spots of adult populations of *An. gambiae *capable of withstanding lethal doses of pyrethroids. This massive elimination of susceptible populations of mosquitoes by spilled petroleum products implicates oil spillage in the selection of pyrethroid resistant populations of *Anopheles *in the south-western Nigeria.

## Authors' contributions

RFD conceived the study and participated in the implementation, data interpretation and manuscript preparation. HSB, ONC contributed in the study design and were fully involved in the implementation of this research and the write up of the manuscript. JMCD contributed in the study design and data analysis. HK, HB, PKOK and MT participated in the design of the study and substantially helped in drafting the manuscript. MCA and AAB guided the study from conception to the manuscript finalization. All authors read and approved the final manuscript.
